# Molecular Characterization and Gene Expression of Glutathione Peroxidase 1 in *Tor tambroides* Exposed to Temperature Stress

**DOI:** 10.1177/1176934319853580

**Published:** 2019-06-13

**Authors:** Thinh Dinh Do, Nguyen Thi Mai, Tran Nguyen Duy Khoa, Ambok Bolong Abol-Munafi, Hon Jung Liew, Chang-Bae Kim, Li Lian Wong

**Affiliations:** 1 Department of Biotechnology, Sangmyung University, Seoul, South Korea; 2 Institute of Marine Environment and Resources, Vietnam Academy of Science and Technology, Haiphong, Vietnam; 3 Institute of Tropical Aquaculture, Universiti Malaysia Terengganu, Kuala Terengganu, Malaysia; 4 College of Aquaculture and Fisheries, Can Tho University Campus II, Cantho, Vietnam; 5 Institute of Marine Biotechnology, Universiti Malaysia Terengganu, Kuala Terengganu, Malaysia

**Keywords:** antioxidant enzymes, thermal stress, gene expression, phylogenetic tree, *Tor tambroides*

## Abstract

Temperature is an abiotic factor that affects various biological and physiological processes in fish. Temperature stress is known to increase the production of reactive oxygen species (ROS) that subsequently cause oxidative stress. Fish is known to evolve a system of antioxidant enzymes to reduce ROS toxicology. Glutathione peroxidase (GPx) family consists of key enzymes that protect fish from oxidative stress. In this study, full-length GPx1 cDNA (GenBank accession no. KY984468) of *Tor tambroides* was cloned and characterized by rapid amplification of cDNA ends (RACE). The 899-base-pair (bp) GPx1 cDNA includes a 576-bp open reading frame encoding for 191 amino acids, plus 28 bp of 5′-untranslated region (UTR) and 295 bp of 3′-UTR. Homology analysis revealed that GPx1 of *T tambroides* (Tor-GPx1) shared high similarity with GPx1 sequences of other fish species. The phylogenetic construction based on the amino acid sequence showed that Tor-GPx1 formed a clade with GPx1 sequences of various fish species. Real-time polymerase chain reaction (PCR) was performed to assess the levels of GPx1 gene expression in the liver and muscle of *T tambroides* under thermal stress. The results indicated that GPx1 gene expression was down-regulated under decreased temperature. However, there was no significant difference between GPx1 gene expression in fish exposed to high temperature and control. Our study provides the first data regarding GPx gene expression in *T tambroides* under thermal stress.

## Introduction

Temperature influences the biological processes of an organism which affect their survivability, but may also determine their distribution in the native ranges.^1,2^ Although climate change is often associated with increased temperature, cold shock catastrophically occurs in natural events such as thermocline temperature variation, abnormal water movements, rapid precipitation, and rapid changes in seasonal temperatures.^3,4^ Fish, like other ectotherms with body temperatures conforming to the environmental temperatures, has metabolic processes highly dependent on water temperature.^5^ Notably, any changes in water temperature could lead to overproduction of reactive oxygen species (ROS) and cause oxidative stress to fish. Indeed, increased ROS level results in lipid peroxidation that potentially damages cellular molecules.^6^ To neutralize the deleterious impact of ROS, fish uses their antioxidant defense mechanisms, which can be either enzymatic or non-enzymatic.^2^ Superoxide dismutase (SOD), catalase (CAT), peroxiredoxin (Prx), and glutathione peroxidase (GPx) are parts of the enzymatic mechanisms involved in the detoxification of ROS.^4,7,8^

Glutathione peroxidases represent an important enzyme family that protects the living organisms from oxidative damage.^8,9^ This family comprises widespread proteins that can be found in most living organisms.^10^ Together with SOD, CAT, and Prx, GPx family is the key enzyme for the detoxification of ROS in aquatic organisms, including fish.^4,11^ Thermal stress may promote the generation of oxidative stress that results in the variation of GPx gene expression levels.^2,12–14^ The complex observation of GPx gene expression can be explained by simultaneous metabolic processes in fish in response to ROS elevation during temperature fluctuations.^14,15^

Glutathione peroxidase enzyme family is divided into 2 groups: the selenium-dependent glutathione peroxidase (Se-GPx), which can reduce both organic and inorganic peroxides, and the selenium-independent glutathione peroxidase (non-Se-GPx), which can reduce only organic peroxide.^16^ In vertebrates, up to 8 distinct GPx isoforms were identified.^17^ Among them, GPx1, GPx2, GPx3, and GPx4 are selenoproteins in mammal, whereas GPx6 is selenoprotein in human only.^18^ In the remaining isoforms, the selenocysteine residue (Sec) is replaced by cysteine.^19^ GPx1 (classical GPx) is first discovered and most abundantly expressed in the GPx family.^20^

Although numerous studies on the enzyme activity of GPx have been performed regarding thermal stress in fish, studies on the gene expression of GPx are limited.^9^ Fish species of the genus *Tor*, commonly referred to as the mahseers, are important to most nations in the Asian region for biodiversity reasons and are also sought after as high-valued food and game fish.^21^ However, overfishing has caused the reduction of *Tor tambroides* populations in the nature.^22^ Given the wide geographical distribution of *T tambroides* (Himalayas to Southeast Asia)^23^ and its capability to survive in a large range of temperature conditions, the temperature tolerance of this species is of particular interest. Taking the possible role of GPx on oxidative stress into consideration, the objectives of this study were to identify GPx sequence and quantify its expression levels in *T tambroides* under temperature transitions. cDNA and amino acid sequences of Tor-GPx1 were characterized and analyzed. Based on full GPx cDNA sequence, we measured the changes in Tor-GPx1 transcript using real-time polymerase chain reaction (PCR) to describe the effects of temperature transitions from 28°C to 2 stress temperature points (11°C and 38°C) on the GPx gene expression.

## Materials and Methods

### Experimental design and sample collection

Fingerlings of *T tambroides* (17.7 ± 1.4 cm; 64.9 ± 16.2 g) were collected from the Freshwater Hatchery of School of Fisheries and Aquaculture Sciences, Universiti Malaysia Terengganu. All experimental procedures of fish were approved by the Ethics Committee of Institute of Tropical Aquaculture, Universiti Malaysia Terengganu. The fish were acclimatized in their respective tanks for a week at 28°C ± 0.5°C and fed to satiation with commercial pellet (TP-2, 28% crude protein, 3% crude fat). After a week of acclimation, fingerlings were randomly selected and distributed into 3 aquaria (250 L). Control temperature was set at 28°C, whereas both upper and lower thermal limits were achieved by increasing 1°C or decreasing 2°C per day from 28°C until the experimental fish displayed signs of extreme heat stress (fading body color, heavy breathing, gasping at the surface for air, and strange swimming behavior) and extreme cold stress (minimal activity, stop swimming, resting at the tank bottom, and increased respiration) during the thermal stress tests. The final experimented temperatures were 11°C, 28°C, and 38°C. Throughout this study, the dissolved oxygen level and water pH were maintained at >5.0 mg L^–1^ and 7.0 ± 0.5, respectively. After the thermal experiment, 8 fish from each tank were randomly collected and dissected. The liver and muscle tissues were preserved in RNA*later* and stored at −20°C prior to total RNA extraction.

### Total RNA extraction and molecular cloning of GPx cDNA

Total RNA was extracted from the liver and muscle tissues using TRIzol (Invitrogen, USA) following the manufacturer’s protocol. The concentration and quality of RNA were measured using ScanDrop 200 (Analytik Jena, Germany). cDNAs were synthesized from 1 µ g total RNA using the iScript Supermix cDNA Synthesis Kit (Bio-Rad, USA).

A fragment of GPx was amplified using degenerate primers by Choi et al.^24^ This fragment was used to design species-specific primers for rapid amplification of cDNA ends (RACE) by Primer3 program.^25^ To obtain full-length sequences of GPx gene for *T tambroides*, RACE was performed using the SMARTer RACE 5′/3′ Kit (Clontech Laboratories, USA). The reaction conditions were as follows: 94°C for 5 minutes, followed by 35 cycles of 94°C for 30 seconds, 68°C for 1 minute, 72°C for 1 minute, and final step of 72°C for 10 minutes. The amplicons were ligated into pRACE vectors and transformed into Stellar Competent Cells (Clontech Laboratories). Plasmid DNA of positive clones was then extracted and purified by Wizard Plus SV Minipreps DNA Purification System (Promega, USA). Three independent clones were sequenced by First BASE Laboratories Sdn Bhd (Malaysia) and showed the same result.

### Sequence alignment and phylogenetic analysis

The open reading frame (ORF) and amino acid sequences of GPx1 were deduced from the full-length cDNA using Geneious v. 9.1.7.^26^ The obtained amino acid sequence was compared with other reported teleost GPx sequences available on National Center for Biotechnology Information (NCBI; www.ncbi.nlm.nih.gov) using BLAST.^27^ GPx1 sequence motifs were identified by MEME Suite.^28^ Amino acid sequences of GPx1 to GPx4 from various vertebrate species were used to construct phylogenetic tree (Supplemental Table S1). The sequences were aligned using Clustal W^29^ and ambiguous positions were excluded using Gblocks.^30^ MEGA X was used to determine the best evolutionary model for the dataset.^31^ The best model was identified as Le and Gascuel^32^ (LG) model together with Gamma (G) distribution of rates. A phylogenetic tree of GPx isoforms was constructed using the maximum-likelihood (ML) method with the LG + G model and 10 000 bootstrap replicates in MEGA X.^31^ Bayesian tree was inferred using MrBayes v3.2.6.^33^ Four independent runs of Markov chain Monte Carlo (MCMC) were performed for 1 000 000 generations, and sampling was done every 1000 generations.

### Expression of GPx1 gene

Real-time PCR was performed to quantify the expression of GPx1 gene in the liver and muscle tissues of *T tambroides*. Amplifications were conducted for triplicates of each sample using the SensiFAST SYBR lo-ROX kit following the standardized protocol (Bioline, USA) in a Mx3005P quantitative polymerase chain reaction (QPCR) system (Agilent technologies, USA). All GPx primers including endogenous control (β-actin gene), which were used in this study, are listed in Table 1.^24,34^ The real-time PCR profile was 10 minutes at 95°C, followed by 40 cycles of 10 seconds at 95°C, 20 seconds at 60°C and 10 seconds at 72°C. The specificity of primer amplification was confirmed by melting curve analysis (60°C-95°C) with a heating rate of 0.5°C/s. To test the amplification efficiency, a standard curve was generated for both targeted and reference genes from a 10-fold serial dilution of cDNA.

**Table 1.** Primer sequences used in this study.

Primer	Sequences (5′ - 3′)	Sources
GPx degenerate primer	F: TACACCCAGATGAACGAGC	Choi et al^24^
	R: AGGAACTTYTCAAAGTTCCAGGA	
GPx1-3′ RACE	F: CTGATCAGGGGCTCGTGGTTCTGGG	This study
GPx1-5′ RACE	R: GTTCGCACCGTTCACTTCCAGCTTCTC	This study
GPx1 real-time PCR	F: GTGACGACTCTGTGTCCTTG	This study
	R: AACCTTCTGCTGTATCTCTTGA	
β-Actin	F: GATGGACTCTGG TGATGGTGTGAC	Xing et at.^34^
	R: TTTCTCTTT CGG CTGTGGTGGTG	

Abbreviations: F, forward; GPx, glutathione peroxidase; PCR, polymerase chain reaction; R, reverse; RACE, rapid amplification of cDNA ends.

β-actin acts as the reference gene.

### Statistical analysis

The GPx1 gene expression levels of all samples were normalized to the expression levels of β-actin gene of the same samples using Relative Expression Software Tool 384 v. 1 (REST).^35^ The transcript levels of fish at 11°C and 38°C were compared and converted to relative fold changes with that at 28°C using the relative quantification method.

## Results

**Figure 1.**
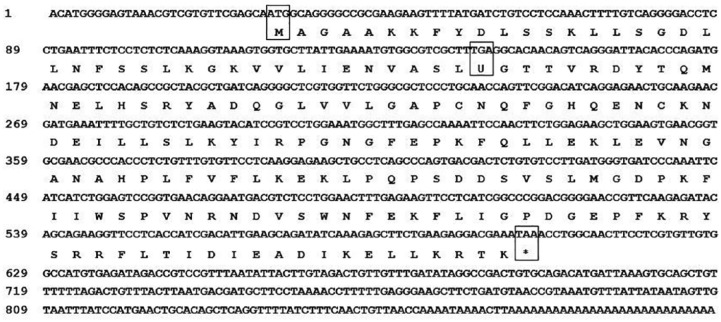
Nucleotide sequence and deduced amino acid sequence of GPx1 cDNA cloned from *Tor tambroides*. The letters in boxes represent the start codon (ATG), selenocysteine codon (TGA), and the stop codon (TAA).

Using the RACE procedure, the full-length cDNA of GPx1 (GenBank accession no. KY984468) was successfully obtained from *T tambroides* (Figure 1). It included 899 bp in length with an ORF of 576 bp, 28 bp of the 5′-untranslated region (UTR), and 295 bp of the 3′-UTR. The ORF was predicted to encode for a protein of 191 amino acids. The ORF contained TGA codon at position 146 (40th codon) encoding for selenocysteine instead of functioning as a stop codon.

**Figure 2.**
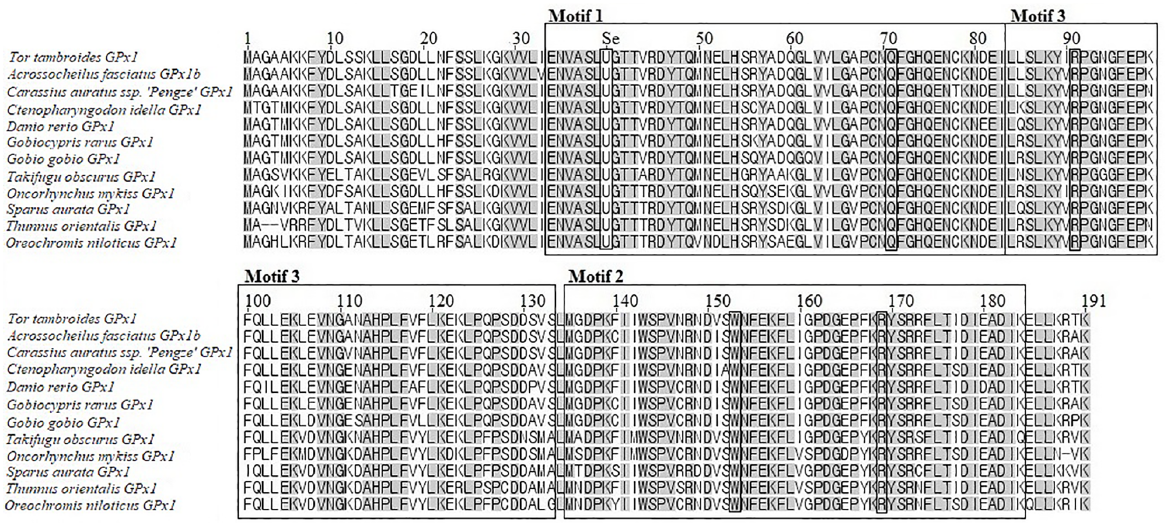
Comparison of deduced amino acid sequence of glutathione peroxidase 1 of *Tor tambroides* with other fishes using Geneious software. Three different boxes present 3 conserved motifs based on the MEME analysis. Selenocysteine (U40) is inserted in the box and labeled as Se. The positions of Glutamine 71 (Q71), Tryptophan 153 (W153), Arginine 91 (R91), and Arginine 169 (R169) are included in the boxes.

The MEME analysis showed that there were 3 signature sequence motifs, each of which includes 50 conserved amino acid residues (Figure 2). Selenocysteine residue (U40) encoded by a stop codon (TGA) is found conserved among all the analyzed fish species. The Tor-GPx1 amino acid sequence was compared with other teleost fish available on GenBank. The results indicated that Tor-GPx1 shared 80% to 96% identity with the GPx sequences of other teleost fish species. Tor-GPx1 was closest to GPx1b of *Acrossocheilus fasciatus*, a cyprinid with 96% similarity (Table 2).

**Table 2.** Sequence identity between *Tor tambroides* GPx1 and other fish GPxs.

Species	GenBank accession no.	Amino acid length	Identity (%)
*Acrossocheilus fasciatus*	AIM56842	191	96
*Carassius auratus* ssp. “Pengze”	AGC50802	191	95
*Ctenopharyngodon idella*	ACF39780	191	93
*Danio rerio*	NP_001007282	191	93
*Gobiocypris rarus*	AHA82628	191	92
*Gobio gobio*	AEX57308	191	91
*Takifugu obscurus*	ACR20471	191	83
*Oncorhynchus mykiss*	CCG28019	190	83
*Sparus aurata*	AFY97790	191	80
*Thunnus orientalis*	CCG28019	189	80
*Oreochromis niloticus*	NP_001266640	191	80

Abbreviation: GPx, glutathione peroxidase.

**Figure 3.**
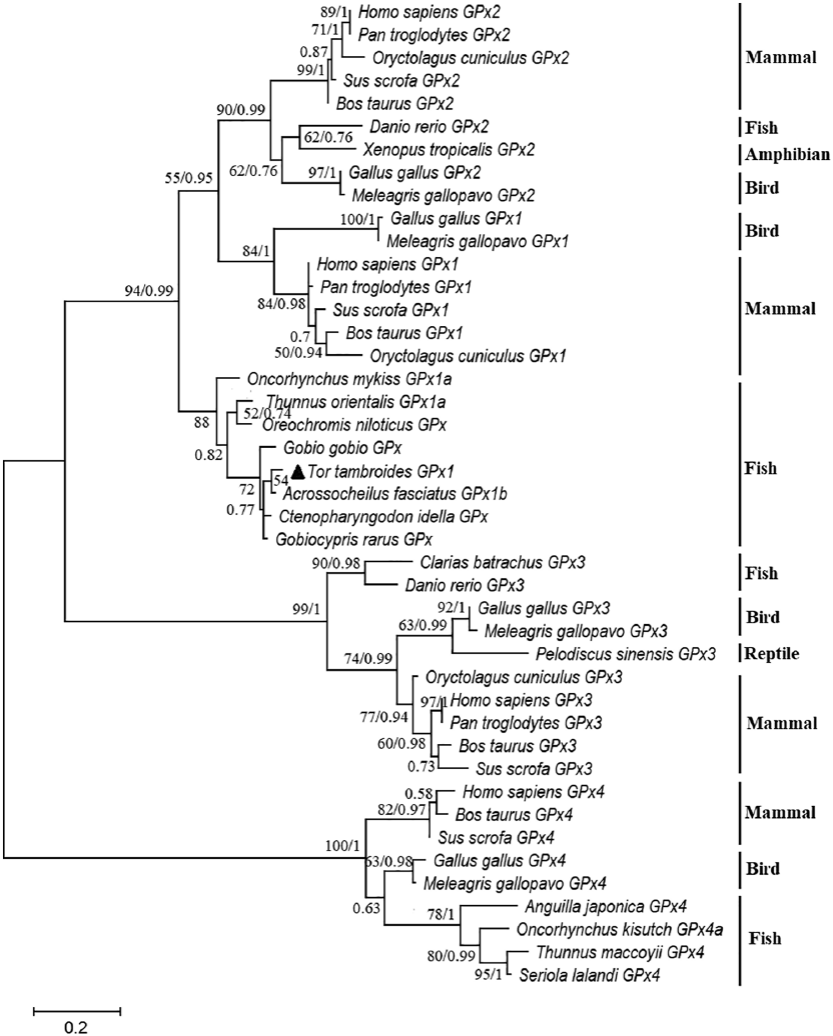
Phylogenetic relationships of *Tor tambroides* and various vertebrate species based on GPx amino acid sequences. The accession numbers of sequences used for phylogenetic analysis are provided in Supplemental Table S1. Bootstrap values > 50 (ML) and probabilities > 0.5 (BI) are shown at the nodes. BI indicates Bayesian inference; GPx, glutathione peroxidase; ML, maximum likelihood.

Maximum-likelihood and Bayesian inference (BI) methods generated phylogenetic trees with consistent topology. A combined tree of GPx sequences from the two methods is presented in Figure 3. Accordingly, there were 3 clusters in the tree, including GPx1/GPx2, GPx3, and GPx4, respectively. Tor-GPx1 formed a clade with other fish’s GPx1 isoforms in the GPx1/GPx2 cluster. Particularly, Tor-GPx1 has a close relationship with GPx1 sequences of other cyprinid species, including *Acrossocheilus fasciatus, Ctenopharyngodon idella, Gobiocypris rarus* and *Gobio gobio*.

**Figure 4.**
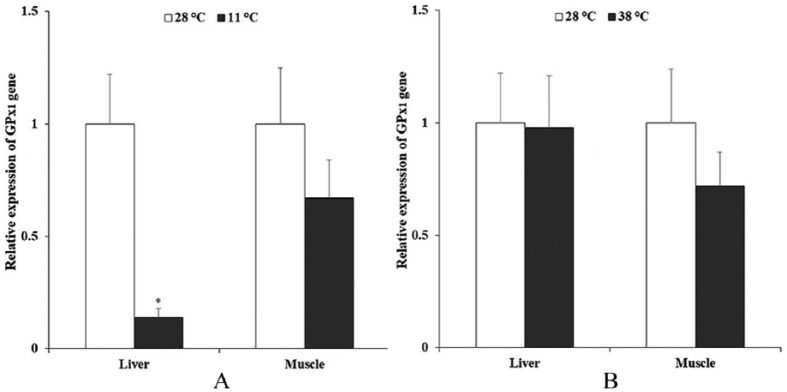
Relative expression of glutathione peroxidase 1 gene (Tor-GPx1) in different tissues of stressed fish and control fish. The RNA expression values of fish exposed to (A) 11°C and (B) 38°C are presented relative to that of control fish at 28°C. Asterisks indicate statistical significance (*P* < .05). GPx indicates glutathione peroxidase.

Figure 4 depicts the relative expression of GPx1 gene in *T tambroides* after exposure to 2 extreme thermal limits, 11°C and 38°C, respectively, compared with the control temperature (28°C). In general, Tor-GPx1 gene expression showed down-regulation at 11°C (Figure 4A). The Tor-GPx1 gene expression in the muscle of fish at 11°C decreased 1.49-fold compared with that at 28°C. Noticeably, Tor-GPx1 gene expression was significantly down-regulated in the liver tissue. The expression level of Tor-GPx1 gene reduced 7.05-fold for individuals at 11°C. In contrast, the gene expression pattern of the Tor-GPx1 gene at 38°C was inherently different from that at 11°C (Figure 4B). There was no significant difference in Tor-GPx1 gene expression at 38°C in comparison with 28°C.

## Discussion

In this study, degenerate primers followed by RACE-PCR were used to obtain the first cDNA sequence of GPx in *T tambroides*. Based on sequence comparison, Tor-GPx1 shows high similarity with other GPx1 sequences in cyprinid species such as *A fasciatus* and *Carassius auratus* ssp. “Pengze.” Three signature sequence motifs with conservation of amino acid were identified (Figure 2). The conservation of amino acid revealed that the essential function of GPx may also be conserved.^36^ Tor-GPx1 is a selenoprotein that includes a selenocysteine amino acid residue. Like other fish, there are conserved amino acid residues in Tor-GPx1 which are related to the role of selenium.^36^ U40 (Selenocysteine 40) is a typical amino acid for selenoprotein and is known to be the catalytic site of the enzyme.^16^ In addition to U40, there are 4 other amino acid residues, which play a crucial role in the enzymatic function of GPx (Figure 2). The first two, Q71 (Glutamine 71) and W153 (Tryptophan 153), are involved in the fixation of selenium.^36,37^ The remaining residues R91 and R169 (Arginine 91 and Arginine 169) are demonstrated with the contribution to direct the donor substrate (glutathione) toward the catalytic center.^36–38^

The homology analysis demonstrated that Tor-GPx1 is similar to GPx1 sequences in fish from GenBank data at different levels. As a member of the cyprinid family, Tor-GPx1 has high similarity with different species in this group. Our results are concordant with previous reports on GPx sequences of cyprinid fishes.^16,36^ A phylogenetic tree was built to investigate the evolutionary relationship between Tor-GPx1 and other GPx isoforms in various vertebrate organisms (Figure 3). The tree indicated that there were 3 clusters: GPx1/GPx2, GPx3, and GPx4. Interestingly, GPx1 of birds and mammals was sister to the GPx2 clade. This result demonstrated a close relationship between GPx1 and GPx2 genes in these organisms. Our finding is in agreement with earlier studies by Malandrakis et al^9^ and Duan et al^39^ based on the phylogenetic relationship of GPx sequences of gilthead sea bream (*Sparus aurata*) and ridgetail white prawn (*Exopalaemon carinicauda*) with that of different animals. As seen from the tree, Tor-GPx1 is closely related to other GPx1 sequences, especially those of fish species (Figure 3). Tor-GPx1 formed a clade with other cyprinid species, showing the conservative character of GPx.^9^ Previous studies reported that there are 2 variants of GPx1 in fish species: GPx1a and GPx1b.^19,36,40^ These 2 variants are found in many fish species of various orders. Protein sequences of 2 variants show high similarity, up to 77.5% in *Larimichthys crocea*^40^ and 81.6% in *A fasciatus*.^36^ High similarity between GPx1a and GPx1b may indicate their similar structure and function.^36^ Two variants may appear through a duplication that occurs early together with the evolutionary process of fish.^19,40^

The identification of Tor-GPx1 sequences allowed us to study its expression under thermal stress. It is well documented that temperature stress induced ROS production that resulted intracellular oxidative stress which triggers antioxidant defense activation as consequences.^41^ GPxs belong to the family of antioxidant enzymes, which catalyzes the reduction of hydrogen peroxide and organic hydroperoxides.^19^ It is reported that GPxs are fairly abundant, widely distributed, and expressed in different organs of fish.^36^ In this study, GPx1 gene expression levels in the liver and muscle of *T tambroides* were investigated within temperature transition. The results did not detect a significant difference in Tor-GPx1 gene expression at an extremely high temperature compared with the control temperature. Several studies have been performed to investigate responses of GPx in fishes to the changes of water temperature. Under elevated temperatures, GPx expression levels in fishes may increase^42,43^ or decrease.^14,44^ Effects of temperature increase on cellular mechanisms are still poorly understood, especially those related to redox chemistry.^45,46^ It is reported that elevated temperature results in enhanced oxygen consumption and ROS production.^42^ In this case, fish need to produce more antioxidant enzymes like GPx to prevent oxidative damages.^47^ On the other hand, a decrease in mitochondrial ROS production (mitochondria are the major sites of ROS production) as a consequence of reduced overall metabolism under thermal stress may result in lower GPx levels.^14,15^ The observation of dissimilar GPx levels among species and tissues under thermal stress demonstrated that GPx expression is tissue specific and species specific.^13^ In the same fish species, acute heat stress and long-term heat stress may show different effects on GPx gene expression. GPx levels in bald notothen (*Pagothenia borchgrevinki*) were unchanged after fish individuals were exposed to a higher temperature for 12 hours, but increased after being exposed to the same temperature for 3 weeks.^45^ The indifferences of GPx gene expression in *T tambroides* under a high temperature compared with control may reflect a similar trend found by Almroth et al^45^ in which GPx gene expression depends on the acute or long-term thermal stress.

Compared with heat shock, fewer studies on the response of antioxidant enzymes have been conducted under cold shock.^4^ In this study, we observed a down-regulation of Tor-GPx1 gene expression when water temperature reduced to 11°C. Our finding corresponds with the reduction of GPx4 gene expression found in common carp (*Cyprinus carpio*).^48^ Similar to heat shock, GPx gene expression in fish exposed to cold shock may depend on organs and exposure time. It is reported that GPx levels in the liver and gill of zebrafish (*Danio rerio*) transferred from 28°C to 11°C showed no changes after 1 hour, but their levels were increased after 6 hours.^4^ However, GPx level in the brain was down-regulated at both 1 and 6 hours.^4^ All these observations elucidated the fact that cold temperature is known to reduce physical activities and metabolic rate in fish.^13^ A decrease in the metabolic rate during cold temperature is reported to reduce the expression of antioxidant genes, including GPx.^41,47,49^ As a consequence, this results in a lower GPx level in fish exposed to cold temperature.

Dissimilar patterns in responses to temperature changes were not only observed in GPx, but also found in other antioxidant genes.^12,13,50^ The inconsistent results in previous studies have been found in different tissues, fish species, temperature acclimation regimes, and/or assays used to assess oxidative stress.^12,50^ When transferring golden fish (*C auratus*) from cold to warm temperatures, increases and decreases in oxidative markers were dependent on the tissues.^51^ Moreover, it is reported that multiple oxidative stress indicators were disturbed as fishes were exposed to hot temperature, but the antioxidant capacity was impacted only to some extent.^15,46^ To accurately evaluate the oxidative stress under temperature changes, both ROS production and responses of antioxidant enzymes should be taken into consideration.

## Conclusions

In this study, we identified full-length GPx1 cDNA and assessed the expression patterns of GPx1 mRNA in the liver and muscle of *T tambroides* in response to temperature changes. Our phylogenetic analysis demonstrated that Tor-GPx1 formed a cluster and had high similarities with other GPx1 sequences from vertebrate species, particularly GPxs of other fishes. Tor-GPx1 contained conserved amino acid residues to function as an antioxidant selenoprotein. The levels of Tor-GPx gene expression showed a downward trend under cold temperature (11°C) and no changes under high temperature (38°C) when compared with the control temperature (28°C). This finding suggests that Tor-GPx1 gene expression was influenced by cold temperature stress which resulted in a reduced metabolic rate.

## Supplemental Material

Table_S1_xyz15512b72a5bee – Supplemental material for Molecular Characterization and Gene Expression of Glutathione Peroxidase 1 in *Tor tambroides* Exposed to Temperature Stress Click here for additional data file. Supplemental material, Table_S1_xyz15512b72a5bee for Molecular Characterization and Gene Expression of Glutathione Peroxidase 1 in *Tor tambroides* Exposed to Temperature Stress by Thinh Dinh Do, Nguyen Thi Mai, Tran Nguyen Duy Khoa, Ambok Bolong Abol-Munafi, Hon Jung Liew, Chang-Bae Kim and Li Lian Wong in Evolutionary Bioinformatics
