# Supplementary material for: Molecular Characterization and Gene Expression of Glutathione Peroxidase 1 in Tor tambroides Exposed to Temperature Stress
Source: Evol Bioinform Online. 2019 Jun 13;15:1176934319853580. doi: 10.1177/1176934319853580 (PMC6572904; doi:10.1177/1176934319853580)
Supplement: Table_S1_xyz15512b72a5bee – Supplemental material for Molecular Characterization and Gene Expression of Glutathione Peroxidase 1 in Tor tambroides Exposed to Temperature Stress [file Table_S1_xyz15512b72a5bee.pdf]

Table S1: The GenBank accession numbers of GPx sequences used for phylogenetic analysis

No.	Species name	GPx isoform	GenBank accession no.
1	*Tor tambroides*	GPx1	KY984468
2	*Acrossocheilus fasciatus*	GPx1b	AIM56842
3	*Ctenopharyngodon idella*	GPx1	ACF39780
4	*Gobiocypris rarus*	GPx1	AHA82628
5	*Gobio gobio*	GPx1	AEX57308
6	*Oncorhynchus mykiss*	GPx1	CCG28019
7	*Thunnus orientalis*	GPx1	CCG28019
8	*Oreochromis niloticus*	GPx1	NP_001266640
9	*Gallus gallus*	GPx1	NM_001277853
10	*Meleagris gallopavo*	GPx1	NM_001308652
11	*Homo sapiens*	GPx1	NP_000572
12	*Pan troglodytes*	GPx1	BAF31850
13	*Bos taurus*	GPx1	NP_776501
14	*Sus scrofa*	GPx1	NP_999366
15	*Oryctolagus cuniculus*	GPx1	NP_001078913
16	*Danio rerio*	GPx2	NP_001316688
17	*Xenopus tropicalis*	GPx2	NM_001256315
18	*Gallus gallus*	GPx2	NM_001277854
19	*Meleagris gallopavo*	GPx2	KR528572
20	*Homo sapiens*	GPx2	NP_002074
21	*Pan troglodytes*	GPx2	NP_001108606
22	*Bos taurus*	GPx2	NP_001156611
23	*Sus scrofa*	GPx2	ABI63991
24	*Oryctolagus cuniculus*	GPx2	NP_001243822
25	*Clarias batrachus*	GPx3	AGI03839

26	*Danio rerio*	GPx3	NP_001131027
27	*Pelodiscus sinensis*	GPx3	JX470527
28	*Gallus gallus*	GPx3	NM_001163232
29	*Meleagris gallopavo*	GPx3	NM_001308655
30	*Homo sapiens*	GPx3	AAH35841
31	*Pan troglodytes*	GPx3	NP_001108629
32	*Bos taurus*	GPx3	AAI49267
33	*Sus scrofa*	GPx3	NP_001108627
34	*Oryctolagus cuniculus*	GPx3	NP_001243829
35	*Oncorhynchus kisutch*	GPx4a	AFD09496
36	*Anguilla japonica*	GPx4	ACN78879
37	*Thunnus maccoyii*	GPx4	ABO38818
38	*Seriola lalandi*	GPx4	AEI91050
39	*Gallus gallus*	GPx4	NM_204220
40	*Meleagris gallopavo*	GPx4	NM_001308651
41	*Homo sapiens*	GPx4	NP_002076
42	*Bos taurus*	GPx4	NP_777195
43	*Sus scrofa GPx*	GPx4	P36968
